# Elevated Plasma SPARC Levels Are Associated with Insulin Resistance, Dyslipidemia, and Inflammation in Gestational Diabetes Mellitus

**DOI:** 10.1371/journal.pone.0081615

**Published:** 2013-12-09

**Authors:** Lu Xu, Fan Ping, Jinhua Yin, Xinhua Xiao, Hongding Xiang, Christie M. Ballantyne, Huaizhu Wu, Ming Li

**Affiliations:** 1 Department of Endocrinology, Key Laboratory of Endocrinology, Ministry of Health, Peking Union Medical College Hospital, Chinese Academy of Medical Sciences and Peking Union Medical College (CAMS & PUMC), Beijing, China; 2 Section of Atherosclerosis and Vascular Medicine, Department of Medicine, Baylor College of Medicine, Houston, Texas, United States of America; 3 Center for Cardiovascular Disease Prevention, Methodist DeBakey Heart Center, Houston, Texas, United States of America; University General Hospital of Heraklion and Laboratory of Tumor Cell Biology, School of Medicine, University of Crete, Greece

## Abstract

**Objective:**

Recent studies suggested that secreted protein acidic and rich in cysteine (SPARC), a novel adipokine, is a key player in the pathology of obesity and type 2 diabetes. We aimed to determine whether concentrations of SPARC were altered in patients with gestational diabetes mellitus (GDM) compared to normal glucose tolerance (NGT) controls and to investigate the relationships between SPARC and metabolic parameters in pregnant women.

**Design/Methods:**

Cross-sectional study of 120 pregnant women with GDM and 60 controls with NGT, in a university hospital setting. Plasma levels of SPARC, adiponectin, fibroblast growth factor 21 (FGF21), insulin and proinsulin were determined by ELISA.

**Results:**

GDM women had higher SPARC and lower adiponectin than NGT subjects; no difference was found in FGF21. SPARC levels were the lowest in subjects in the third tertile of insulin sensitivity index (ISI_OGTT_) and correlated positively with pre-pregnant BMI, insulin and 3 h glucose during 100-g OGTT, HOMA-IR, fasting proinsulin, hsCRP and white blood cells count, and negatively with ISI_OGTT_, when adjusting for gestational age. Triglyceride (TG), Apolipoprotein A1, apolipoprotein B and lipoprotein (a) correlated with SPARC in partial Pearson correlation. Correlations between SPARC with adiponectin, systolic blood pressure and TG were marginally significant in partial Spearman correlation analysis. In multivariate regression analysis, SPARC was an independent negative indicator of ISI_OGTT_.

**Conclusions:**

SPARC levels are correlated significantly with inflammation and may also be correlated with dyslipidemia and represent an independent determinant of insulin resistance in late pregnancy, indicating a potential role of SPARC in the pathophysiology of GDM.

## Introduction

Gestational diabetes mellitus (GDM) has been defined as any degree of glucose intolerance with onset or first recognition during pregnancy [1]. GDM prevalence has increased by ∼10–100% in several race/ethnicity groups during the past 20 years [2]. A true increase in the prevalence of GDM, aside from its adverse consequences for infants in the newborn period, might also reflect or contribute to the current patterns of increasing diabetes and obesity, especially in the offspring [3].

Although the mechanisms for the development of GDM are unclear, similar underlying pathophysiology have been proposed for GDM and type 2 diabetes, including insulin resistance and relative insulin deficiency due to failure of pancreatic beta cells [4]. Importantly, in the past decade, a growing body of evidence has identified two pathologic changes implicated in insulin resistance and beta cell dysfunction: inflammation of adipose tissue and dysregulation of adipokine [5], [6]. For example, hypoadiponectinemia and increased levels of leptin, IL-6 and TNF-α have each been found to contribute to insulin resistance or beta cell failure in GDM [7], [8]._ENREF_8_ENREF_9.

Recently, secreted protein acidic and rich in cysteine (SPARC), has been suggested as a key player in the pathology of obesity and type 2 diabetes. SAPRC, also known as osteonectin or BM40 [9], is a widely expressed profibrotic protein with pleiotropic functions. As a modulator of cell-surface interaction, SPARC modulates tissue physiology by altering cell–ECM interactions, cell proliferation and migration [10]. Lately, it was found that adipocytes are the major source of circulating SPARC and SPARC inhibits adipogenesis and promotes adipose tissue fibrosis [11], [12]. Moreover, increased SPARC expression in adipose tissue is associated with insulin resistance. Clinical studies also revealed an association of increased SPARC levels with T2DM and diabetic retinopathy and nephropathy [13], [14]. Animal studies with *Sparc*-knockout mice showed amelioration of T2DM and its complications in the absence of SPARC [15], supporting a causal role of SPARC in the development of T2DM and its complications. Another novel adipokine is fibroblast growth factor 21 (FGF21), which has been reported to have beneficial effects on glucose homeostasis and insulin sensitivity in animal studies [16], [17], and reports on FGF21 in GDM were limited and controversial [18], [19].

Unlike other adipokines such as adiponectin and leptin, circulating levels of SPARC have not been reported so far in GDM. Based on this fact and the roles of SPARC in insulin resistance and obesity-related disease, we sought to examine whether circulating levels of this adipokine are altered in GDM women compared to control subjects and to investigate a potential link between levels of SPARC with clinical and biochemical measures of glucose, lipid metabolism, insulin sensitivity and inflammation in these subjects.

## Subjects and Methods

### Ethics Statement

Informed written consent was obtained from each participant and the study protocol was approved by the Institutional Review Board of Peking Union Medical College Hospital (PUMCH).

### Subjects

Study subjects were recruited from the PUMCH Pregnant Cohort as described elsewhere [20]. Briefly, all pregnant women in outpatient were subjected to a standardized 50 g glucose challenge test (GCT) between the 24^th^ to 28^th^ gestational weeks. Subjects with 1 h plasma glucose level <7.8 mmol/L were defined GCT negative (GCT^–^); Subjects with 1 h plasma glucose level ≥7.8 mmol/L were considered GCT positive (GCT^+^) and underwent an additional oral glucose tolerance test (OGTT) with 100 g glucose 1 week later. Diagnosis of GDM was made based on the ADA criteria[1], i.e., if ≥1 of the following were present, GDM was diagnosed: the plasma glucose values ≥5.3 mmol/L on fasting; 10.0 mmol/L at 1 h; 8.6 mmol/L at 2 h; and 7.8 mmol/L at 3 h after oral glucose challenge. Normal glucose tolerance (NGT) was diagnosed when plasma glucose levels were below the values at all time points. We randomly selected 120 GDM patients (from a total of 723 women with GDM) and 60 age–matched normal controls (from a total of 582 women with NGT), with a total of 180 pregnant women, for this study. Patients with pre-gestational diabetes mellitus, treatment with steroid hormones, serious systemic disorders such as lupus, congenital cardiopathy, chronic hepatitis and nephrosis were excluded.

### Anthropometric measurement

Anthropometric indices of the patients were collected at the first prenatal examination between the 13^th^ and 15^th^ weeks of gestation and included age, height, pre-pregnancy weight, history of gravidity and parity, systolic and diastolic blood pressure, past history, family history of diabetes (first degree relatives). Pre-pregnancy body mass index (pre-BMI) was calculated as weight (kg)/height (m)^2^.

### Blood samples and biochemical analysis

After overnight fast, venous blood samples were collected from each subject by direct venipuncture into tubes with or without EDTA as anticoagulant. Serum and plasma were obtained by centrifugation at 1000 x g for 20 min. White blood cells (WBC) count and serum levels of creatinine (Cr) and alanine transaminase(ALT) were measured using standard laboratory procedures at the first prenatal examination between the 13^th^ and 15^th^ weeks of gestation. In the second trimester of pregnancy (24–28^th^ weeks of gestation), additional blood chemistry were obtained as follows: plasma glucose levels were determined by the glucose oxidize method; serum levels of triglyceride (TG) and total cholesterol (TC) were determined using standard enzymatic method; serum levels of high density lipoprotein cholesterol (HDL-C) and low density lipoprotein cholesterol (LDL-C) were measured by direct method using the Hitachi 7060C Automatic Biochemistry Analysis System (Tokyo, Japan). Serum apolipoprotein A1 (ApoA1), apolipoprotein B (ApoB), lipoprotein (a) (Lp(a)) and hypersensitive C reaction protein (hsCRP) were measured using Beckman's turbidimetric immunoassay method(CA, USA). Plasma glycosylated hemoglobin (HbA1c) levels were determined by high performance liquid chromatography (HPLC) method using Bio-Rad's VariantIITURBO analyzer (CA, USA).

### Oral glucose tolerance test (OGTT)

OGTTs were performed in the second trimester of pregnancy. The gestational age when OGTTs were performed was presented and used for data analysis. After overnight fast, the subjects took 100 g glucose orally. At 0 h, 1 h, 2 h, and 3 h after glucose intake, plasma glucose (mmol/L) and specific insulin (*u*IU/mL) concentrations were measured as described previously [20]. Insulin resistance was assessed by homeostasis model assessment (HOMA) (HOMA-IR = [fasting insulin]×[fasting glucose]/22.5) and insulin sensitivity was assessed by Matsuda and DeFronzo's insulin sensitivity index (ISI_OGTT_ = 10,000/√([fasting plasma glucose×fasting plasma insulin]×[mean glucose×mean insulin]) [21]. In an earlier validation study in pregnant women IS_OGTT_ exhibited better correlation with insulin sensitivity derived using the euglycaemic–hyperinsulinaemic clamp technique than did the HOMA-IR [22].

### Determination of plasma levels of SPARC, adiponectin, FGF21 and proinsulin

Fasting plasma samples collected in the second trimester of pregnancy were frozen at −80°C for later adipokines analysis. Plasma levels of proinsulin, adiponectin, SPARC and FGF21 were measured by sandwich ELISA. Proinsulin and adiponectin were measured as described previously with intra- and interassay coefficient of variations (CVs) of <8.9%, <11.2, <5.4 and <8.5%, respectively [23]–[25]. FGF21 was measured by an ELISA kit (Phoenix Pharmaceuticals, Burlingame, CA, USA) with intra- and interassay CVs of <6.0% and <8.6% respectively. For SPARC assay, mouse anti-human SPARC monoclonal antibody ON1-1 (Invitrogen, CA, USA) at 0.025 ug/well and biotinylated polyclonal goat anti-human SPARC antibody EYR01 (R&D Systems, MN, USA) at 0.015 ug/well were used. The plasma samples were diluted at 1∶20 with 10% Block Ace (Dainippon Pharmaceutical, Osaka, Japan). In this ELISA system, human SPARC HON-3030 (Haematological Technologies Inc., Vermont, USA) was used to generate the standard curve to quantify the SPARC protein based on absorbance data. The intra- and interassay CVs were 5.2% and 9.1%, respectively. All samples were tested in duplicates in a blinded manner.

### Statistical analysis

SPSS software version 13.0 (SPSS, Chicago, IL) and SAS software version 9.2 (SAS Institute Inc, Cary, NC) were used for statistical analyses. To evaluate the differences of the characteristics between two groups, independent sample Student *t* test was applied. General linear model was used to adjust pre-BMI in comparison. Univariate correlations between SPARC and metabolic variables in pregnancy were assessed both by partial Pearson and partial Spearman correlation analysis with adjustment of gestational age. To identify independent relationships between parameters and dependent variable of Ln ISI_OGTT_, multivariate stepwise linear regression analysis was performed. Variables that were associated with Ln ISI_OGTT_ in univariate correlation analyses were selected into the model as independent variables, including pre-BMI, gestational age, systolic blood pressure, triglycerides, SPARC, adiponectin, proinsulin, hsCRP, WBC count and ALT. Distribution was tested for normality using Shapiro-Wilk W test, and non-normally distributed parameters were logarithmically transformed, where necessary. A p value < 0.05 was considered statistically significant in all analyses.

## Results

### Plasma levels of SPARC and FGF21 in subjects with NGT and GDM

Table 1 showed the baseline demographic, clinical and metabolic characteristics of the study population in pregnancy, stratified into two groups: NGT (n = 60) and GDM (n = 120). Women in the two groups were matched for age and had similar pre-BMI, gestational age and blood pressures.

**Table 1:** Anthropometric and metabolic characteristics of the subjects.

	NGT(n = 60)	GDM(n = 120)	*p* value
Age(yrs)	30.5 (28.0–35.0)	32.0 (29.0–34.0)	0.189
Gestational age (wks)	26.6 (25.1–27.8)	26.2 (24.9–27.5)	0.141
pre-BMI(kg/m^2^)	20.4 (19.2–22.0)	21.5 (19.8–23.0)	0.089
Systolic blood pressure (mm Hg)	112 (105–120)	110 (105–120)	0.858
Diastolic blood pressure (mm Hg)	66 (61–70)	68 (60–74)	0.616
Glucose 0 h(mmol/L)	4.5 (4.2–4.8)	4.8 (4.5–5.1)	<0.001***
Glucose 1 h(mmol/L)	8.0 (7.2–8.9)	10.1 (9.3–11.1)	<0.001***
Glucose 2 h(mmol/L)	7.2 (6.5–7.6)	9.0 (7.9–9.8)	<0.001***
Glucose 3 h(mmol/L)	6.6 (5.9–7.0)	7.8 (7.1–8.6)	<0.001***
Insulin 0 h (*u*IU/mL) ^a^	6.3 (4.7–8.6)	7.8 (5.5–10.7)	0.028*
Insulin 1 h (*u*IU/mL) ^a^	66.2 (35.3–106.9)	90.7 (52.1–133.6)	0.001**
Insulin 2 h (*u*IU/mL) ^a^	65.4 (44.4–106.9)	120.0 (62.7–152.6)	<0.001***
Insulin 3 h (*u*IU/mL) ^a^	53.2 (37.8–74.3)	92.8 (54.3–129.6)	<0.001***
ISI_OGTT_^a^	11.1 (7.2–15.3)	6.7 (5.1–9.7)	<0.001***
HOMA-IR ^a^	1.2 (0.9–1.7)	1.7 (1.2–2.3)	0.007**
Fasting proinsulin(pmol/L) ^a^	9.8 (7.6–12.2)	12.5 (8.4–14.4)	0.012*
ALT(U/L) ^a^	15.0 (10.8–32.2)	19.0 (13.0–40.0)	0.060
Cr (*u*mol/L)	60.2 (57.5–63.7)	61.0 (57.0–65.0)	0.600
TG (mmol/L) ^a^	2.24 (2.00–2.60)	2.51 (1.99–3.14)	0.040*
TC(mmol/L)	6.21 (5.43–6.61)	5.95 (5.17–6.74)	0.406
HDL-C(mmol/L)	2.22 (1.98–2.45)	2.02 (1.81–2.38)	0.246
LDL-C(mmol/L)	3.58 (3.13–4.08)	3.39 (2.73–3.92)	0.143
ApoA1(g/L) ^a^	1.6 (0.9–2.4)	0.9 (0.5–1.4)	<0.001***
ApoB(g/L) ^a^	1.2 (0.7–1.5)	1.9 (1.2–2.5)	<0.001***
Lp(a) (mg/L) ^a^	180.0 (75.0–279.3)	145.5 (76.5–312.5)	0.591
White blood cells (x10^9^/L)	9.1 (7.7–10.3)	10.7 (9.0–11.9)	0.030*
hsCRP(mg/L) ^a^	1.9 (0.9–3.6)	3.0 (1.6–4.6)	0.033*
Adiponectin(ug/mL) ^a^	14.6 (8.3–18.9)	10.5 (4.2–16.5)	<0.001***
SPARC(ng/mL) ^a^	146.4 (73.1–221.9)	151.7 (103.4–274.2)	0.040*
FGF21(ng/mL) ^a^	0.3(0.1–0.9)	0.2 (0.1–0.8)	0.282

Data were shown as median (interquartile range). Independent sample T test was used to compare characteristics between two groups. ^a^ Skewed distributions were logarithmically transformed for comparison.* P<0.05, **P<0.01, ***p<0.001. pre-BMI, pre-pregnancy body mass index; ISI_OGTT_: insulin sensitivity index; HOMA-IR, HOMA of insulin resistance index; ALT, alanine transaminase; Cr, Creatinine; TG, triglyceride; TC, total cholesterol; HDL-C, high density lipoprotein cholesterol; LDL-C: low density lipoprotein cholesterol; ApoA1, apolipoprotein A1; ApoB, apolipoprotein B; Lp(a), lipoprotein (a); hsCRP, hypersensitive C reaction protein; SPARC, secreted protein acidic and rich in cysteine; FGF21 fibroblast growth factor 21.

GDM women had higher levels of glucose and insulin at all time points during 100 g OGTT than NGT subjects (p<0.05). As glucose tolerance status worsened, HOMA-IR and fasting plasma level of proinsulin increased in GDM patients compared with NGT women(p = 0.007, p = 0.012, respectively). Similarly, some lipids (TG, p = 0.040; ApoB, p<0.001) and inflammatory markers (hsCRP, p = 0.033; WBC, p = 0.030) were higher in GDM than in NGT. Values of ISI_OGTT_ and ApoA1 showed the opposite pattern, being lower in GDM than in NGT women (p<0.001 for both). Serum levels of ALT, Cr, TC, HDL-C, LDL-C and Lp(a) exhibited no differences between the two groups.

Plasma concentrations of SPARC were significantly increased (p = 0.040) and adiponectin were markedly decreased (p<0.001) in GDM patients compared to NGT subjects. The group differences of SPARC remained significant after adjustment of pre-BMI (p = 0.022, data not shown). We failed to find a difference in plasma FGF21 levels between the two groups.

**Figure 1:**
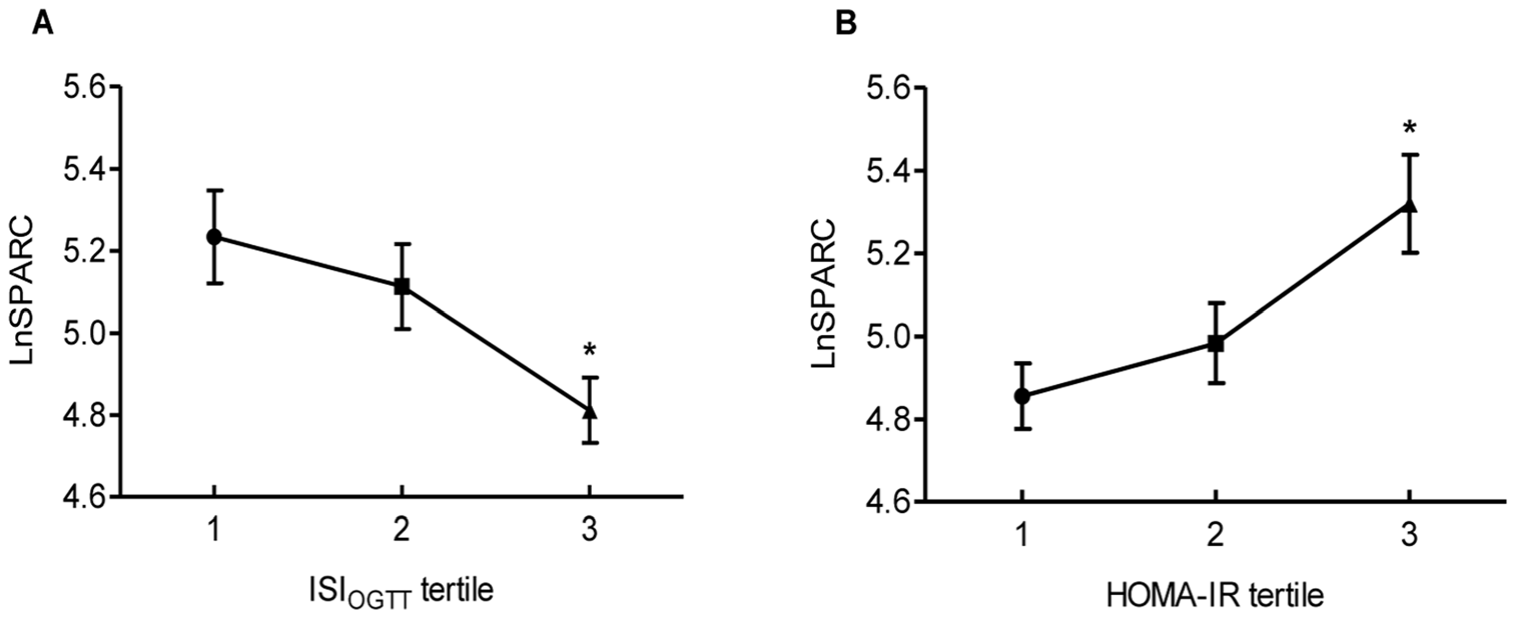
A. LnSPARC in ISI_OGTT_ tertiles; B. LnSPARC in HOMA-IR tertile. Data were shown mean±SEM. SPARC were logarithmically transformed for comparison. Differences between groups were assessed by One-way ANOVA. SPARC plasma levels are decreased from first to third tertile of ISI_OGTT_ and increased from first to third tertile of HOMA-IR. **A**. * p = 0.003, T3 vs T1; p = 0.033, T3 vs T2; **B**. *p = 0.001 T3 vs T1; p = 0.017, T3 vs T2.

When patients were divided by extent of insulin sensitivity into ISI_OGTT_ tertiles, plasma levels of SPARC were lower in the second and third tertile as compared with the first tertile, and the differences between the third and the first tertile (p = 0.003) as well as the third and the second tertile (p = 0.033; Fig. 1A) were significant. Accordingly, SPARC levels increased progressively from the first to the third tertile of HOMA-IR (p = 0.001, T3 vs T1; p = 0.017, T3 vs T2; Fig. 1B).

### Univariate correlations

As SPARC is expressed and secreted by placental tissue we adjusted for gestational age in the univariate correlation analysis. Partial Pearson correlation analysis after adjustment showed that plasma SPARC concentrations were positively associated with pre-BMI (r = 0.153, p = 0.040), insulin resistance: 3-hour glucose (r = 0.222, p = 0.003), 0-,1-,3 hours insulin during 100-g OGTT (r = 0.220, p = 0.003; r = 0.175, p = 0.018; r = 0.177, p = 0.017; respectively), HOMA-IR (r = 0.219, p = 0.003), fasting plasma levels of proinsulin (r = 0.182, p = 0.015), lipids including TG (r = 0.151, p = 0.045), ApoB (r = 0.153, p = 0.040) and Lp(a) (r = 0.168, p = 0.025), and inflammatory markers such as hsCRP (r = 0.175, p = 0.018) and WBC count(r = 0.203,p = 0.007; Table 2); whereas significant negative correlations existed between SPARC and ISI_OGTT_ (r = −0.236, p = 0.001), SPARC and ApoA1(r = −0.159, p = 0.034). In contrast, circulating SPARC levels were not significantly correlated with age, blood pressure, 0-, 1-, 2-hours glucose and 2-hour insulin during 100-g OGTT, ALT, Cr, TC, HDL and LDL cholesterol and plasma adiponectin and FGF21 (Table 2) in Partial correlation analysis.

**Table 2:** Univariate correlations between SPARC and metabolic parameters.

	partial Pearson correlation analysis #		partial Spearman correlation analysis #	
	r	p	r	P
FGF21(ng/mL) ^a^	0.096	0.216	0.066	0.375
Adiponectin (mg/L) ^a^	0.094	0.219	−0.126	0.090^§^
Age (yrs)	−0.121	0.106	−0.106	0.157
Pre-BMI(kg/m^2^)	0.153	0.040*	0.167	0.024*
Systolic blood pressure (mm Hg)	0.122	0.127	0.147	0.064^§^
Diastolic blood pressure (mm Hg)	0.060	0.452	0.101	0.206
Glucose 0 h (mmol/L)	0.090	0.226	0.102	0.171
Glucose 1 h (mmol/L)	0.103	0.167	0.112	0.132
Glucose 2 h (mmol/L)	0.026	0.729	0.006	0.940
Glucose 3 h (mmol/L)	0.222	0.003*	0.229	0.002*
Insulin 0 h (*u*IU/mL) ^a^	0.220	0.003*	0.259	<0.001*
Insulin 1 h (*u*IU/mL) ^a^	0.175	0.018*	0.193	0.009*
Insulin 2 h (*u*IU/mL) ^a^	0.108	0.150	0.115	0.125
Insulin 3 h (*u*IU/mL) ^a^	0.177	0.017*	0.191	0.011*
Fasting proinsulin(pmol/L) ^a^	0.182	0.015*	0.185	0.014*
HOMA-IR ^a^	0.219	0.003*	0.251	0.001*
ISI_OGTT_^a^	−0.236	0.001*	−0.245	0.001*
ALT(U/L) ^a^	−0.031	0.694	−0.025	0.748
Cr (*u*mol/L)	0.085	0.276	0.099	0.201
TG (mmol/L) ^a^	0.151	0.045*	0.129	0.086^§^
TC (mmol/L)	−0.039	0.609	−0.073	0.330
HDL-C (mmol/L)	−0.101	0.173	−0.084	0.265
LDL-C (mmol/L)	0.019	0.804	−0.060	0.428
ApoA1(g/L) ^a^	−0.159	0.034*	0.071	0.343
ApoB(g/L) ^a^	0.153	0.040*	0.065	0.389
Lp(a)(mg/L) ^a^	0.168	0.025*	0.084	0.263
White blood cells (×10^9^/L)	0.203	0.007*	0.236	0.002*
hsCRP (mg/L) ^a^	0.175	0.018*	0.189	0.011*

# Univariate correlation analyses were adjusted for gestational age. * p<0.05; ^§^ 0.05≤p≤0.1. ^a^ Skewed distributions were logarithmically transformed for partial Pearson correlation analysis. SPARC, secreted protein acidic and rich in cysteine; FGF21 fibroblast growth factor 21; pre-BMI, pre-pregnancy body mass index; ISI_OGTT_: insulin sensitivity index; HOMA-IR, HOMA of insulin resistance index; ALT, alanine transaminase; Cr, Creatinine; TG, triglyceride; TC, total cholesterol; HDL-C, high density lipoprotein cholesterol; LDL-C: low density lipoprotein cholesterol; ApoA1, apolipoprotein A1; ApoB, apolipoprotein B; Lp(a), lipoprotein (a); hsCRP, hypersensitive C reaction protein.

In accordance with the results of partial Pearson analysis, in partial Spearman correlation analysis adjusted for gestation age, SPARC levels positively correlated with pre-BMI (r = 0.167, 0.024; Table 2), 3-hour glucose (r = 0.229, p = 0.002), 0-,1-,3 hours insulin during 100-g OGTT(r = 0.259, p<0.001; r = 0.193, p = 0.009; r = 0.191, p = 0.011; respectively), HOMA-IR (r = 0.251, p = 0.001), fasting plasma levels of proinsulin (r = 0.185, p = 0.014), inflammatory markers including hsCRP (r = 0.189, p = 0.011) and WBC count(r = 0.236,p = 0.002); and negatively related to ISI_OGTT_ (r = −0.245, p = 0.001). Differently from results of Pearson correlation analysis, SPARC lost its correlations with lipoprotein ApoA1, ApoB and Lp (a) in Spearman analysis, but showed marginally significant correlation with adiponectin (r = −0.126, p = 0.090), systolic blood pressure (r = 0.147, p = 0.064) and TG (r = 0.129, p = 0.086).

### Multiple regression analysis

Having demonstrated that GDM is characterized by elevated level of SPARC, which in turn related to insulin resistance, we next queried whether the relationship between SPARC and insulin resistance is independent of the other metabolic parameters. Multivariate stepwise regression analysis with logarithmically transferred ISI_OGTT_ as a dependent variable was applied to investigate the relationship. SPARC (β = −0.156, p = 0.030), adiponectin (β = 0.295, p<0.001), fasting proinsulin (β = −0.365, p<0.001), hsCRP (β = −0.236, p = 0.001) and TG (β = −0.162, p = 0.032) were independently associated with insulin sensitivity as estimated by ISI_OGTT_ (Table 3).

**Table 3:** Multivariate stepwise linear regression analysis with Ln insulin sensitivity index (ISI_OGTT_) as dependent variable.

Independent variable	Beta coefficient	Standard error	t	p value
Fasting proinsulin ^a^	−0.365	0.065	−5.225	<0.001
Adiponectin ^a^	0.295	0.006	4.008	<0.001
hsCRP ^a^	−0.236	0.017	−3.268	0.001
TG ^a^	−0.162	0.058	−2.171	0.032
SPARC ^a^	−0.156	0.052	−2.201	0.030

Independent variables included in the model were pre-BMI, gestational age, systolic blood pressure, triglycerides, SPARC, adiponectin, fasting proinsulin, hsCRP, WBC count and ALT. ^a^ Skewed distributions were logarithmically transformed for analysis. ISI_OGTT_: insulin sensitivity index; hsCRP, hypersensitive C reaction protein; TG, triglyceride; SPARC, secreted protein acidic and rich in cysteine.

## Discussion

In our study, we first determined circulating levels of SPARC in pregnant women and found that SPARC levels were elevated significantly in GDM group compared with NGT group and correlated significantly with insulin resistance. We also demonstrated the associations between SPARC with inflammatory markers, TG, and adiponectin. Importantly, the key finding of the current study was the demonstration that SPARC in pregnancy was an independent indicator of insulin resistance. In agreement with this, SPARC level was significantly lower in subjects in the third tertiles of ISI_OGTT_ as compared with the first tertile. Therefore, our findings implicate a potential role of SPARC in the pathophysiology of insulin resistance in GDM and provide insights on both risk stratification and modification in this patient population.

So far there are limited studies on SPARC and diabetic subjects, and the findings are consistent with our results: levels of SPARC are significantly elevated in T2DM patients compared with normal controls in Chinese and Japanese populations [14], [26]. A recent study showed that human placenta villi could express and secrete SPARC [27], suggesting that levels of SPARC may be affected by gestational age. However, in our study the subjects in GDM group and control group had similar gestational age, around 26 weeks of gestation and it has been shown that circulating levels of SPARC mainly come from adipose tissue [11], therefore, the differences in circulating levels of SPARC in GDM patients and controls may not be caused by gestational age. To avoid the potentially confounding effect of gestational age, we still adjusted for it in our univariate and multivariate correlations.

In the above studies about diabetic subjects, SPARC positively correlate with BMI, the percentage of fat, fasting insulin and 2 h insulin in OGTT, HOMA-IR and triglyceride in T2DM and normal controls [14], [26]. The correlations of levels of SPARC and levels of insulin at different time points during OGTT in these studies and our study may be explained by the interaction between SPARC and insulin. On the one hand, insulin may increase SPARC production, as evidenced by in vitro study where insulin promotes SPARC expression of visceral adipose tissue explants in a dose-dependent manner [11]. On the other hand, over-expression of human SPARC in INS-1 cells increases glucose-stimulated insulin secretion [28]. The influence of SPARC in insulin resistance may also arise from its role in obesity-induced adipose tissue fibrosis and associated metabolic dysfunction by regulating extracellular matrix (ECM) composition and inhibiting adipogenesis [29], [30]. Fibrosis of subcutaneous adipose tissue may reduce the ability of adipocytes to store triglycerides, which then overspill into the circulation, resulting in systemic hyperlipidemia and, ultimately, lipid infiltration into other organs such as skeletal muscle and liver leading to ectopic lipid deposition and insulin resistance in these organs. The implication of SPARC in dysfunction of adipose tissue could also explain our findings that SPARC were positively related to level of triglycerides but negatively with adiponectin. However, the correlation between SPARC and ApoA1, ApoB and Lp (a) were not consistent in Pearson correlation analysis and Spearman correlation analysis in our study, indicating that SPARC and these TG rich lipoprotein maybe linear related rather than monotonic related. Taken together, the association of SPARC and insulin resistance may be mediated by dysfunction of adipose tissue and related dyslipidemia. As increasing evidence has shown that levels of ApoB, especially when combined with apoAI to form the ApoB/A1 ratio, have higher sensitivity and specificity than cholesterol as predictors of atherosclerotic cardiovascular disease [31], and TG is an important component of metabolic syndrome [32], our results also suggested risk of elevated SPARC in GDM for future cardiometabolic disorders.

A growing body of evidence has recently reported that women with GDM exhibit evidence of sub-clinical inflammation. For example, increased CRP levels and leukocyte count in the first trimester have been demonstrated to independently predict the subsequent development of GDM later in pregnancy [33], [34]. In our study, for the first time we showed that circulating SPARC levels in the second trimester positively correlated with hsCRP levels and also associated with WBC count that was detected in the earlier trimester of pregnancy. A previous study also indentified strong correlation between serum hsCRP and SPARC expression in adipose tissue [11]. SPARC may link inflammation and glucose intolerance by excessive synthesis of ECM components [35]. A proinflammatory environment up-regulates expression of SPARC which then promotes the synthesis of ECM components and drives adipose tissue fibrosis and subsequent insulin resistance. The co-existence of high SPARC level and elevated inflammation markers in GDM and their close relevance with each other and with insulin resistance suggest that their interaction may play an important role in the development and progression of GDM.

In the current study, GDM was diagnosed based on the criteria of the American Diabetes Association as long as glucose value at any time point during OGTT exceeds its threshold. In central Europe, the use of the more stringent criteria for the diagnosis of GDM women detects more large-for-gestational age neonates with hypoglycemia and mothers with impaired postpartum glucose metabolism than the World Health Organization criteria [36]. Furthermore, Kautzky-Willer and coworkers [36] demonstrated convincingly that women with 1 abnormal glucose value during OGTT do not differ from those with 2 abnormal values in their obstetric outcome. According to this stricter criteria used in our study, adiponectin levels were significantly different in GDM group compared with NGT group, and low adiponectin emerged as an independent contributor to insulin resistance in pregnant women. Low adiponectin has been repeatedly associated with insulin resistance, consistent with the known insulin-sensitizing bioactivity of the protein [37]. This inverse relationship between adiponectin and insulin resistance has also been observed in GDM [38]. Thus, our results on adiponectin were in accordant with the previous findings of its protected role in development of insulin resistance in pregnancy, indicating its beneficial effects in Chinese pregnant women.

We did not find a significant difference in FGF21 between GDM and NGT women. So far studies on FGF21 in GDM were limited and controversial. Stein et al. assessed the levels of FGF21 in German GDM women at mid-pregnancy (24–28^th^ week of gestation) and showed that serum FGF21 levels were not significantly different between subjects with GDM and healthy pregnant controls [18]. While another recent study in UK in GDM women at 39–40 weeks of gestation reported that GDM women had significantly higher plasma levels of FGF21 than controls [19]. In regard to the role of FGF21 in lipid metablism and insulin resistance during pregnancy, the current study highlights the need for future study in this population of different races and ethnics.

Our current study included some limitations. Firstly, given the cross-sectional nature of our study, temporal associations were not determine. Secondly, due to the complicated procedures of hyperinsulinemic-euglycemic clamp, it is impractical for use in large populations. Therefore, we did not use this method in the evaluation of insulin resistance in our study, but we used multiple simple indices including HOMA-IR, fasting proinsulin and ISI_OGTT_, which consistently shown the correlation between SPARC and insulin resistance. Finally, because of the difficulty in obtaining informed consent, we could not detect circulating SPARC levels in women with normal 50 g OGTT and compare SPARC levels between GCT^–^ subjects and GDM subjects.

In conclusion, our results revealed that elevated plasma level of SPARC in GDM correlated with inflammatory markers and dyslipidemia and more importantly, was independently associated with insulin resistance in pregnant women, suggesting a potential role of SPARC in the pathogenesis of GDM, and hence may be relevant to strategies for both risk stratification and risk modifications in pregnant women.
